# Dietary Intervention Improves Gastrointestinal Symptoms after Treatment of Cancer in the Pelvic Organs

**DOI:** 10.3390/jcm12144766

**Published:** 2023-07-19

**Authors:** Mette Borre, Janne Fassov, Jakob Lykke Poulsen, Peter Christensen, Søren Laurberg, Asbjørn Mohr Drewes, Klaus Krogh

**Affiliations:** 1 Department of Hepatology and Gastroenterology, Aarhus University Hospital, Palle Juul-Jensens Boulevard 99, 8200 Aarhus, Denmark; jannfass@rm.dk (J.F.); klaukrog@rm.dk (K.K.); 2 Danish Cancer Society Centre for Research on Survivorship and Late Adverse Effects after Cancer in the Pelvic Organs, Aarhus University Hospital, 8200 Aarhus, Denmark; japo@rn.dk (J.L.P.); petchris@rm.dk (P.C.); soeren.laurberg@aarhus.rm.dk (S.L.); amd@rn.dk (A.M.D.); 3 Mech-Sense, Department of Gastroenterology and Hepatology, Aalborg University Hospital, 9000 Aalborg, Denmark; 4 Department of Surgery, Aarhus University Hospital, Palle Juul-Jensens Boulevard 99, 8200 Aarhus, Denmark

**Keywords:** cancer, sequelae, colorectal, pelvic, complications, diet, dietary intervention

## Abstract

Gastrointestinal (GI) symptoms are common in patients receiving radiotherapy, chemotherapy, and/or surgery for cancer in the pelvic organs. The aim of the present prospective cohort study was to report the efficacy of dietary intervention in patients with chronic GI sequelae to treatment of cancer in pelvic organs and insufficient symptomatic effect of medical treatment. Eighty-eight patients were offered specialist dietitian guidance. Gastrointestinal symptoms and quality of life were assessed before and after intervention by validated questionnaires. The main dietary interventions were low-fat diet (n = 44; 50%), modification of dietary fiber content (n = 19; 33%), dietary restrictions with a low-FODMAP (fermentable oligosaccharides, disaccharides, monosaccharides, and polyols) diet (n = 18; 20%), gluten-free diet (n = 1; 1%), and other dietary advice (n = 6; 7%). Compared to baseline, dietary intervention improved quality of life (EQ5D scale) (*p* < 0.01), bowel function for the last four weeks (*p* < 0.02), stool frequency (*p* < 0.03), constipation (*p* < 0.05), incomplete rectal emptying at defecation (*p* < 0.02), and performing usual activities (*p* < 0.0). In conclusion, this observational study using tailored dietary intervention showed that symptoms can be reduced and quality of life can be improved in patients with chronic GI sequelae following treatment of cancer in the pelvic organs not responding sufficiently to medical treatment.

## 1. Introduction

Chronic adverse effects, including gastrointestinal (GI) symptoms, are common in patients who receive radiotherapy, chemotherapy, and/or surgery for cancer in the pelvic organs. Thus, 25% to 63% of patients treated suffer from chronic GI symptoms and report that their quality of life (QoL) is negatively affected due to these complications [1,2,3,4,5]. Urgency for defecation, fecal incontinence, fragmented stool, increased bowel frequency, chronic diarrhea, pain, and flatulence are the most common symptoms [5]. Loose stools, urgency, and fecal incontinence are characteristic following right-sided hemicolectomy for colon cancer [6,7]. In about 80%, this is due to bile acid malabsorption (BAM) having resected parts of the terminal ileum [8]. Furthermore, resection of the ileocecal valve may allow retrograde passage of colonic content, resulting in small intestinal bacterial overgrowth (SIBO) and dysfunction of bile acid circulation [8]. In contrast, constipation, difficult emptying, and incomplete rectal evacuation are the main symptoms in patients treated with a left-sided colon resection [9]. Partial and total mesorectal excision in patients with rectum cancer can cause low anterior resection syndrome mainly characterized by fecal incontinence, increased bowel frequency, and urgency for defecation [10,11]. Moreover, pelvic radiotherapy may result in bile acid malabsorption (BAM) and chronic diarrhea [4,5] in patients treated for prostate, cervical, and anal cancer. As for chemotherapy, long-term effects on bowel function remain to be studied in detail with regard to the specific chemotherapy agent. In right-sided hemicolectomy patients receiving chemotherapy, late adverse gastrointestinal symptoms do not seem to be affected by the chemotherapy [6,9], but BAM is a well-known complication in leukemia patients treated with chemotherapy [12,13] and may cause changes in gut microbiota [5]. 

The most well-described factors contributing to gastrointestinal symptoms after treatment of cancers in the pelvic organs are SIBO and BAM [5,8]. Lactose intolerance may occur from pelvic radiotherapy and chemotherapy due to reduction in brush border enzymes [14]. 

The complex nature of bowel symptoms after treatment of cancer in pelvic organs warrants systematic assessment and management. In BAM, the bile acid sequestrants cholestyramine or colesevelam are recommended first-line medical treatment [14,15]. However, not all patients have sufficient symptom relief, and a combination of bile acid sequestrant and low-fat diet may further reduce symptoms [14,15,16,17]. Furthermore, a low-fat diet may be considered as a first-choice treatment in patients with mild BAM or in cases where urgency and fecal incontinence are prominent although stool consistency is normal [15,16]. Symptomatic SIBO is normally treated with oral antibiotics, of which rifaximin is the most well documented. Nevertheless, one study has shown that 80% of patients with SIBO had symptom relief following an elemental diet for 14 days [18]. Moreover, carbohydrate intolerance (e.g., lactose, fructose, and fructans) is very common among SIBO patients, and dietary restrictions with a low FODMAP (fermentable oligosaccharides, disaccharides, monosaccharides, and polyols) diet might lead to symptomatic improvement [19]. 

Cancer survivors are usually encouraged to follow common lifestyle recommendations with potential beneficial effects on general health and recurrence of the disease. However, there is a lack of consensus behind the dietary information given, evidence is weak, and some dietary advice may even aggravate symptoms. [14,20]. Only 13 out of 51 guidelines for treatment and follow-up of colon cancer have recommendations for managing late sequelae, and here a variety of dietary advice is given [21,22]. 

We hypothesized that individual counselling by a specialized dietitian would be of major benefit in patients with chronic complications to treatment cancer of in the pelvic organs. The aim of the present study was to report the efficacy of targeted dietary intervention as an add-on to optimized medical treatment in such patients. 

## 2. Materials and Methods

### 2.1. Design

In this prospective cohort study performed at a tertiary center at the Department of Hepatology and Gastroenterology at Aarhus University Hospital, Aarhus, Denmark from April 2018 to December 2021, we consecutively included patients unsatisfied with current bowel function and remaining GI symptoms despite medical treatment for specialist dietitian guidance. The study was registered in the Central Denmark Region’s register of research projects (no. 1-16-02-972-17).

### 2.2. Patients

All patients were survivors of a cancer in the pelvic organs treated with surgery and/or radio-/chemotherapy. Patients had been referred to the Department of Hepatology and Gastroenterology at Aarhus University Hospital, Aarhus, Denmark for specialist evaluation by a gastroenterologist (JF) specialized in treatment of GI sequelae to treatment of cancer. During dietary intervention, patients continued taking their usual medication, including medication prescribed by the gastroenterologist before referral to the dietitian, but no other medication affecting gut function was added. 

### 2.3. Clinical and Demographic Characteristics

For the present study, data were prospectively collected after medical treatment before dietary intervention, and again 8–12 weeks after dietary intervention. Symptoms had to be present for at least 6 months. Information regarding type of cancer and additional oncological or surgical treatment were collected from patients’ charts. Nutritional status, weight, height, and body mass index (BMI) were assessed at baseline and at follow-up with the dietitian. 

### 2.4. Clinical Evaluation

The main criteria for assessment of severity of bowel dysfunction was the patient’s own global assessment of bowel function within the last four weeks. The following other chronic bowel symptoms were registered: daily number of bowel movements (>3 bowel movements per day were defined as frequent), stool consistency defined by the Bristol Stool Chart (with type 6 and 7 defining loose stools) [23], urgency for defecation, fecal incontinence, nocturnal defecation, abdominal bloating [10,24], and impact of bowel function on quality of life. The validated EuroQol five-dimensional five-level (EQ-5D-5L) questionnaire was used to evaluate quality of life [25]. The questionnaires were answered electronically at home.

As part of standard evaluation before treatment in the clinic, blood samples for celiac disease, primary lactose intolerance, thyroid disease, and malabsorption were performed, and a fecal sample for calprotectin to exclude inflammatory bowel disease was performed. Patients with diarrhea had a breath test for hydrogen and methane performed in order to determine the presence of SIBO and a selenium-75 homocholic acid taurine (SeHCAT) scan to detect BAM. SeHCAT retention levels <15% were considered diagnostic for BAM, and borderline BAM was defined as retention of 16–20% [16]. Patients having had resection of the small intestine or cholecystectomy were not offered SeHCAT as previous data have shown that they most likely have BAM [26].

### 2.5. Dietary Intervention

Dietary intervention comprised two to five individual sessions with the dietitian either as supplement or as an alternative to medical treatment with insufficient symptom relief, immediately after the physician considered that all relevant medical treatment options had been tried. In addition to the primary dietary intervention, which typically is a low-fat diet, further modification of the diet using, e.g., a moderate-low-FODMAP diet or a modification of the fiber content was allowed by discretion of the dietitian. If a patient was diagnosed with BAM and SIBO, the primary dietary intervention was normally a low-fat diet. If a patient was diagnosed with celiac disease or lactose intolerance, the dietary intervention was a primary treatment according to standard principles. All patients were seen by the same dietitian (MB). 

In all cases, the dietary intervention was supported by individual written diet sheets and oral suggestions to alternative food items for the patients. Patients’ partners or family were also invited to the consultation in order to improve adherence to treatment. Follow-up sessions with the dietitian were arranged after 4 weeks and again at 8–12-week intervals when needed. Due to either long distance to the hospital or restrictions related to the COVID-19 pandemic, some patients had consultations and follow-up by telephone or video.

A seven-day diet diary and a modified food frequency questionnaire (FFQ) concerning the intake of fiber- and fat-containing food items, e.g., questions concerned the use of dairy products, beverages, bread, cold cuts, garnish, fats, e.g., spread on bread and fats for cooking, portion sizes, and type of products were completed before the first session with the dietitian. In accordance, the daily average content of fat and fiber were calculated, and the dietitian was able to demonstrate to each patient the sources of fat, fiber, fermentable carbohydrates (FODMAPs), or lactose, etc., in their diet. All food diaries were analysed by one dietitian (MB). Based on main symptoms and, if possible, underlying pathophysiology, the following dietary interventions were made:

### 2.6. Dietary Intervention against Bile Acid Malabsorption

Patients with a SeHCAT scan less than 15%, or who had undergone small intestinal resection or cholecystectomy, were offered bile acid sequestrants (cholestyramine and colesevelam) as a first-line treatment. If they then still had residual symptoms considered to be due to bile acid malabsorption, or if they had significant side effects to the bile acid sequestrants, a low-fat diet was instituted. A low-fat diet was the first-line treatment (i.e., before medication) in patients with borderline BAM (SeHCAT 16–20%). Hence, these patients were advised on how to reduce their fat intake so that their daily fat dietary intake did not exceed 20% of their calculated total energy requirements—usually a maximum of 30–50 g fat per day. 

Patients with both BAM and SIBO were first treated with antibiotics and then bile acid sequestrants. If symptoms persisted, the primary dietary intervention was a low-fat diet. If further modification of the diet was needed, a moderate-low-FODMAP diet was recommended.

### 2.7. Dietary Intervention against SIBO

Patients with SIBO were treated with antibiotics (ciprofloxacin 500 mg BID for 7 days and rifaximin 550 mg BID for 6 days) as a first-line treatment. Those still having residual symptoms were offered dietary intervention to reduce the content of fermentable carbohydrate (a moderate low FODMAP diet). 

Some patients with SIBO and signs of either constipation or diarrhea were also offered dietary intervention changing (increase/reducing) the amount of fiber in their diet, if indicated by their food diary.

### 2.8. Symptom Based Dietary Intervention

Patients with diarrhea not related to BAM or SIBO, where symptoms were not relieved by loperamide or psyllium, were offered dietary intervention reducing the amount of fiber or FODMAPs in their diet. Contrarily, patients with constipation were offered dietary intervention normally increasing the amount of fiber in their diet and maybe reducing, but not totally excluding, the content of fermentable carbohydrate (FODMAPs). 

### 2.9. Dietary Adherence

Dietary adherence was not formally assessed, but all patients seen by the dietitian were asked about adherence, the dietary effects on bowel function, and difficulties in following the dietary advice. 

### 2.10. Data Analysis

Data are presented as numbers (%) or medians with interquartile range (IQR) unless otherwise indicated. Symptoms at baseline and after dietary intervention were analyzed as ordinal data using the Wilcoxon signed-rank test. The paired sample *t*-test was used to compare EQ-5D score at baseline and after dietary intervention. All reported *p*-values are two-tailed and values less than 0.05 are considered statistically significant. Data were analyzed using Stata version 17.0 (StataCorp LLC, College Station, TX, USA) [27]. 

## 3. Results

We included 88 patients (age 55–72, median 61, 57 females) who received dietary intervention as a supplement or an alternative to medical treatment with insufficient effect on GI symptoms. Most patients (n = 63) had been treated for colorectal cancer. Time since completion of treatment for cancer was 6–240 months (mean 53). Among the 88 patients, 32 had undergone surgery alone, 6 had surgery and radiation therapy, 6 had radiation therapy alone, 19 had surgery and chemotherapy, 14 had surgery with chemo- and radiation therapy, 10 had chemo- and radiation therapy, and in 1 the treatment was unknown. Mean (SD) daily fat intake, before dietary intervention, was 79 (19) g per day, fat energy percentage was 38 (6), and mean daily fiber intake was 20 (9) g per day (Table 1).

The patients’ previous cancer diagnosis, main symptoms, and underlying pathology are shown in Table 1 and Table 2. Most patients had BAM and/or SIBO. Thus, 33 patients had bile acid retention <20% on SeHCAT scan and another 13 were considered as having BAM due to previous ileal resection or cholecystectomy. Of these 46 patients (52%) diagnosed with BAM, 26 (56%) patients were also diagnosed with SIBO. A total of 58 (66%) patients had SIBO and GI symptoms despite treatment with antibiotics. Patients with SIBO received no antibiotics during the study period.

Primary dietary interventions are listed in Table 3. Depending on underlying diagnosis and dietary intake, the following diets were commenced: low-fat diet in 44 (50%), low-FODMAP diet in 18 (21%), modification of dietary fiber content in 19 (21%), gluten-free diet in 1 (1%), and other dietary advice in 6 (7%) patients. Depending on residual symptoms and dietary intake, a low-FODMAP diet and/or an increase or a reduction in fiber intake was recommended as an additional intervention in 33 (38%). For response to dietary intervention, see Table 2. Overall, we observed improvement of the following outcomes: bowel function for the last four weeks, quality of life (EQ5D scale), frequency of stools, incomplete rectal emptying at defecation, and performance of usual activities. Looking at patients with SIBO and BAM separately, overall assessment of bowel function, EQ5D, frequency of stools, and incomplete rectal emptying improved in patients with SIBO. In patients with BAM, performance of usual activities improved, while improvement in overall assessment of bowel function and stool consistency were only of borderline significance (*p* = 0.05). In patients with both SIBO and BAM, we observed improvement in stool consistency (*p* < 0.01), frequency of stools (*p* < 0.03), and EQ5D (*p* < 0.02).

## 4. Discussion

This prospective cohort study is, to our knowledge, the first to report results of dietary intervention as a supplement to medical treatment of symptoms in patients with late GI adverse effects after treatment of cancer in the pelvic organs. Our main finding is that tailored dietary intervention relieves a range of symptoms and improves quality of life.

### 4.1. Structured Management vs. Trial and Error

Observational studies have indicated an association between diet and bowel symptoms, and many cancer survivors are left with dietary and behavioral changes based on trial and error [28]. Thus, 32% of colon cancer survivors report that food affects their bowel function. Especially, fat or spicy food items were perceived to cause symptoms [28]. Earlier studies on colorectal cancer survivors have shown that changes in dietary habits were associated with improved quality of life, but unfortunately no assessment of bowel symptoms was performed [29]. In the present study, GI symptoms and the EQ-5D questionnaire were systematically registered at patients´ entrance to the clinic (baseline), after end of medical treatment of GI symptoms, and again after dietary intervention. It has previously been shown that a low-fat diet can reduce diarrhea in cancer survivors with BAM [13,16]. However, reflecting on clinical practice, the dietary intervention in the present study was individually tailored depending on underlying diagnosis, e.g., BAM or SIBO, but also based on the patient´s normal dietary intake, weight status, and ability to follow a diet. All food diaries were analyzed by the same dietitian, which has been shown to improve reliability of the information obtained [30], and instructions were supported by individual written diet sheets and oral suggestions to alternative food items. Starting dietary interventions, follow-up sessions can be essential to ensure adherence to intervention, and in the present study patients had between two to five follow-up sessions.

### 4.2. Recommendations for Dietary Interventions

Cancer survivors are often encouraged to follow general lifestyle recommendations with potential beneficial effects on general health and recurrence of the disease, without recognizing that such advice may negatively affect bowel symptoms. Hence, they are usually recommended a diet high in fiber from vegetables, fruits, and whole grains, chicken and fish, and less refined sugars, fats, and red or processed meat [31,32]. However, the high content of fiber might initiate or worsen diarrhea in some patients and symptoms associated with excess air production in others [14]. In a former study from our group, about 10% of patients reported that vegetables and fruits caused discomfort while another 20% found them helpful [12]. In this study, 21% of patients were treated with a modification of dietary fiber intake. We do not know if patients in the present study had tried dietary interventions before entering the study, but previous studies report that 27% of cancer survivors independently seek information about diet and nutrition in relation to their previous disease or current GI symptoms [12]. Motivation is pivotal for dietary intervention. All our patients had severe GI symptoms before the dietary intervention was commenced and they all adhered to and completed a seven-day diet diary before the first meeting with the dietitian. Our study did not assess the placebo response from dietary intervention, but based on experience from other patient groups, it is probably high [33]. 

### 4.3. Dietary Treatment in Patients with Bile Acid Malabsorption

The mainstay treatment for BAM is bile acid sequestrants [13,15,16]. Unfortunately, not all patients have sufficient symptom relief and others are unable to tolerate treatment [34]. Previous prospective studies among patients with BAM have demonstrated significant improvements in symptoms from a low-fat diet. In these studies, total fat was reduced to 20% of daily energy intake [16]. In theory, a low-fat diet seems a simple and attractive choice, but caution should be taken in patients with low body weight or poor oral intake. None of our patients had a low dietary intake or low body mass index (BMI). Furthermore, some patients may have concerns about losing weight as this may include a fear of recurrence of their cancer [35]. In patients who had only BAM and no other gastrointestinal diagnoses, the improvement in symptoms were only of borderline significance except for usual activities. In a study from Gupta et al. [16], patients with a sole diagnosis of BAM reported the greatest improvement in symptom score. In the study by Gupta et al., the dietary fat intake decreased from an initial mean of 62 g per day to 42 g per day after dietary intervention for BAM. In our study, the mean baseline dietary fat intake among patients instructed to follow a low-fat diet was much higher, 84 g per day, and the corresponding mean fat energy was 38%. In a national survey of the dietary intake of fat in the Danish population between 18–75 years, the intake of fat was 96 g per day and mean fat energy was 36% [36]. We did not formally calculate the content of fat in our patients’ diets during or after intervention. Instead, dietary adherence was measured through self-reporting, which might be a problem for adherence. Our patients were advised on how to reduce their fat intake so that their daily fat dietary intake did not exceed 20% of their calculated total energy requirements—usually a maximum of 30–50 g fat per day. Since our patients’ dietary fat intake initially was high, even a moderate reduction in fat intake presumably would be enough to reduce the bowel symptoms, but the change in symptoms was not significant. The initial fat intake was much higher than in the study by Gupta et al. and probably more difficult to change for our patients. Patients face barriers and facilitators for dietary compliance. Some main factors include practicality of making a lifestyle change, lack of motivation, social circumstances, negative perception of the new diet, and difficulty changing well-established habits [37]. One of the most impactful barriers is losing the ability to eat foods that an individual previously enjoyed [38]. 

### 4.4. Dietary Treatment in Patients with Small Intestinal Bacterial Overgrowth

Small intestinal bacterial overgrowth causes a constellation of symptoms including bloating, abdominal discomfort, gas, and diarrhea. Treatment of SIBO remains empirical and usually includes broad-spectrum antibiotics. However, evidence for their use is generally low and the recurrence rate of symptoms is high [19]. Given the non-specific nature of symptoms, the inability to predict which antibiotic may be effective against which constellation of symptoms, and the potential for development of drug resistance, there is a call for other treatment strategies. Some patients with SIBO may have symptoms from lactose, not due to enzyme deficiency, but due to premature exposure of lactose to bacteria in the small bowel [39]. This effect may also be true for small fermentable carbohydrates like fructose, oligosaccharides, polyols (FODMAPs), and sucrose. Previous studies have observed that a low-FODMAP diet may affect the gut microbiota [19]. Furthermore, an elemental diet formulation consisting of completely hydrolyzed nutrients normalizes SIBO in a greater proportion of patients than antibiotics [18]. The mechanism is unclear but could include rapid absorption and assimilation of the elemental formulation with little available substrate for the bacteria. The total daily intake of FODMAPs in a habitual diet differs between countries and is estimated to range from 15 g to 30 g per day [40]. The amount of FODMAPs in a typical Danish diet is 30 g per day [41] and high compared to data from UK (21–30 g), Sweden (21 g), and Spain (21 g) [42]. We did not formally calculate the content of FODMAPs in our patients’ habitual diets but gave suggestions depending on their daily intake of food known to include FODMAPs. Due to the complexity of a low-FODMAP diet, a moderate-low-FODMAP diet was chosen, e.g., by reducing the content of rye bread, onion, apple, chickpeas, and kidney beans. We did not perform a control breath test for hydrogen and methane after dietary intervention. However, we found that patients treated for SIBO with a low-FODMAP diet reported an improvement in overall assessment of bowel function and quality of life. However, the role of dietary intervention in patients with SIBO needs further evaluation [19]. 

### 4.5. Patients with Combined Diagnosis

Reflecting on clinical practice, about one-third of our patients had more than one diagnosis, e.g., SIBO and BAM. This poses a challenge in management as well as interpreting and presenting data. These patients followed a low-fat diet for 4–6 weeks before evaluation of symptoms. If symptom relief was considered insufficient, patients were instructed to add a low-FODMAP diet. Also, they were occasionally recommended provocation with food either containing high FODMAPs or high fat. For most patients, this resulted in aggravation in GI symptoms. 

### 4.6. Limitations

The present study was observational in design. A randomized controlled trial would have been preferable, but very difficult to conduct on dietary intervention and with several possible interventions. Major limitations are that we did not register adherence to diet changes and no long-term follow-up was performed. We did not collect data on socioeconomic status or the ethnic composition of our study population. Denmark is, however, a very homogeneous society with a healthcare system almost entirely funded by the government. Hence, the population treated very much reflects the entire population of cancer patients and the vast majority would be Caucasian. There was no evaluation between the primary dietary intervention and the second dietary intervention. 

## 5. Conclusions

In conclusion, with targeted dietary intervention, several symptoms and quality of life can be improved in patients with late GI sequelae following treatment of cancer in the pelvic organs. Our study supports the use of dietary intervention as a supplement to medical treatment of GI symptoms. The dietary guidance should be individually tailored depending on the patients’ main symptoms and underlying pathophysiology. 

## Figures and Tables

**Table 1:** Baseline characteristics of the patients.

Gender, males n (%)	31 (35)
Age, median (IQR), years	61 (55–72)
Weight, median (IQR), kg	78 (66–92)
BMI, mean (SD)	26.2 (21.9–29.4)
Previous cancer diagnosis	
Colon	41
Rectal	22
Anal	7
Prostate	6
Cervical	7
Ovarian	1
Other	4
SIBO *, n tested/positive (% tested positive) Bile acid malabsorption	79/58 (73)
	46 (52)
Yes	41
Borderline	5
No	42
Dietary variables	
Daily fat intake, mean (SD), g	79 (19)
Fat energy percentage, mean (SD), %	38 (6)
Fiber intake, mean (SD), g	20 (9)

* Small intestinal bacterial overgrowth, positive for either hydrogen, methane, or both.

**Table 2:** Median symptom score before and after dietary intervention for all patients, SIBO, and BAM, respectively.

	All			SIBO			BAM		
	Before	After	*p*-Value	Before	After	*p*-Value	Before	After	*p*-Value
Bowel function last 4 weeks	4	3	0.02 *	4	3	0.01 *	3	3	0.05 *
EQ5D scale ^#^	61	66	0.01 *	61	68	0.01 *	64	69	0.10
Alteration in lifestyle	3	3	0.53	4	3	0.49	4	4	0.22
Stool frequency <1 per day 1–3 per day 4–7 per day >7 per day	0 40 33 11	0 43 26 7	0.03 *	0 24 22 9	0 29 18 5	0.02 *	0 19 19 4	0 20 16 2	0.10
Stool consistency	5	5	0.93	4.5	5	0.42	5	5	0.05 *
Abdominal pain	3	3	0.98	3	3	0.82	2	3	0.98
Pain relief	2	2	0.75	2	2	0.96	2	2	0.90
Bloating	3	4	0.40	3	3	0.26	3	3	0.50
Passing gas	4	4	0.35	4	4	0.12	4	3	0.23
Constipation	2	2	0.05 *	2	2	0.08	2	2	0.93
Diarrhea	3	2	0.18	2	2	0.11	3	2	0.17
Urgency	5	4	0.07	4.5	3.5	0.05 *	5	4.5	0.25
Incomplete emptying	4	3	0.02 *	4	3	0.01 *	3.5	2.5	0.16
Long fullness	2	2	0.80	2	1	0.42	1.5	1	0.30
Usual activities	2	2	0.04 *	2	2	0.10	2	1	0.02 *
Nocturnal defecation	2	2	0.54	2	2	0.19	2	2	0.95
Fecal incontinence—loose stools	1	1	0.33	1	1	0.14	2	1	0.12

* All reported *p*-values were two-tailed, and values less than 0.05 were considered significant. ^#^ mean. SIBO: small intestinal bacterial overgrowth; BAM: bile acid malabsorption.

**Table 3:** Primary dietary intervention.

	All (n = 88)	SIBO (n = 58)	BAM (n = 46)
Fat-reduced diet	44	30	41
Moderate low FODMAP	18	12	2
Fiber increase	16	10	1
Fiber reduction	3	3	0
Gluten-free diet	1	1	1
Other dietary advice	6	2	1
